# A Jak1/2 inhibitor, baricitinib, inhibits osteoclastogenesis by suppressing RANKL expression in osteoblasts *in vitro*

**DOI:** 10.1371/journal.pone.0181126

**Published:** 2017-07-14

**Authors:** Kohei Murakami, Yasuhiro Kobayashi, Shunsuke Uehara, Takako Suzuki, Masanori Koide, Teruhito Yamashita, Midori Nakamura, Naoyuki Takahashi, Hiroyuki Kato, Nobuyuki Udagawa, Yukio Nakamura

**Affiliations:** 1 Department of Orthopedic Surgery, Shinshu University School of Medicine, Matsumoto, Nagano, Japan; 2 Institute for Oral Science, Matsumoto Dental University, Shiojiri, Nagano, Japan; 3 Department of Biochemistry, Matsumoto Dental University, Shiojiri, Nagano, Japan; Charles P. Darby Children's Research Institute, 173 Ashley Avenue, Charleston, SC 29425, USA, UNITED STATES

## Abstract

The Janus kinases (Jaks) are hubs in the signaling process of more than 50 cytokine or hormone receptors. However, the function of Jak in bone metabolism remains to be elucidated. Here, we showed that the inhibition of Jak1 and/or Jak2 in osteoblast-lineage cells led to impaired osteoclastogenesis due to the reduced expression of receptor activator of nuclear factor-κB ligand (RANKL). Murine calvaria-derived osteoblasts induced differentiation of bone marrow cells into osteoclasts in the presence of 1,25-dihydroxyvitamin D_3_ (1,25D_3_) and prostaglandin E_2_ (PGE_2_) *in vitro*. However, treatment with the Jak1/2 inhibitor, baricitinib, markedly inhibited osteoclastogenesis in the co-culture. On the other hand, baricitinib did not inhibit RANKL-induced osteoclast differentiation of bone marrow macrophages. These results indicated that baricitinib acted on osteoblasts, but not on bone marrow macrophages. Baricitinib suppressed 1,25D_3_ and PGE_2_-induced up-regulation of RANKL in osteoblasts, but not macrophage colony-stimulating factor expression. Moreover, the addition of recombinant RANKL to co-cultures completely rescued baricitinib-induced impairment of osteoclastogenesis. shRNA-mediated knockdown of Jak1 or Jak2 also suppressed RANKL expression in osteoblasts and inhibited osteoclastogenesis. Finally, cytokine array revealed that 1,25D_3_ and PGE_2_ stimulated secretion of interleukin-6 (IL-6), IL-11, and leukemia inhibitory factor in the co-culture. Hence, Jak1 and Jak2 represent novel therapeutic targets for osteoporosis as well as inflammatory bone diseases including rheumatoid arthritis.

## Introduction

Osteoclasts are bone-resorbing cells that differentiate from monocyte-macrophage lineage cells [1]. This differentiation is tightly regulated by osteoblast lineage cells such as osteoblasts [2] and osteocytes [3, 4]. Osteoblasts express two essential cytokines for osteoclast differentiation: receptor activator of nuclear factor-κB ligand (RANKL) and macrophage colony-stimulating factor (M-CSF) [1]. The expression of RANKL is induced by bone resorption factors including 1,25-dihydroxyvitamin D_3_ (1,25D_3_) and prostaglandin E_2_ (PGE_2_) [1]. RANKL binds to its receptor RANK in osteoclast precursors and induces expression of nuclear factor-activated T cell cytoplasmic 1 (NFATc1), which is a master transcription factor for regulating terminal differentiation of osteoclasts [5].

The Janus kinases (Jaks) are a family of intracellular tyrosine kinases that function as hubs in the signaling process of more than 50 cytokine or hormone receptors [6, 7]. The mammalian Jak family has four members, named Jak1, Jak2, Jak3, and tyrosine kinase 2 (Tyk2), which selectively bind different receptor chains [8]. The selective usage of Jak by different receptors yields specific or relatively discrete functional outcomes. Therefore, Jaks have been demonstrated to be excellent targets for therapeutic interventions for various cytokine-mediated disorders.

Cytokine signaling *via* Jaks also plays an important role in osteoclast formation [9]. To date, several studies have investigated whether Jak inhibition can influence osteoclast formation. Tofacitinib (a pan-Jak inhibitor) had no effect on RANKL-induced osteoclast differentiation [10, 11]. On the other hand, Jak2 inhibition with AG490 suppressed osteoclast differentiation induced by RANKL [12, 13]. However, it remains unclear which Jak plays a role in osteoclastogenesis, or whether inhibition of Jak influences osteoblasts’ ability to regulate osteoclast formation.

In the current study, we demonstrate that a selective Jak1 and Jak2 inhibitor, baricitinib, inhibits osteoclastogenesis by suppressing *RANKL* expression in osteoblasts induced by 1,25D_3_ and PGE_2_ *in vitro*. Adenovirus-mediated knockdown of Jak1 or Jak2 also inhibits osteoclast formation. These findings indicate that baricitinib is a potential therapeutic agent to prevent bone resorption.

## Material and methods

### Mice

Male ddY mice, aged 6 weeks, were purchased from Japan SLC (Hamamatsu, Shizuoka, Japan). Animal experiments were performed in compliance with the 3Rs, and all efforts were made to minimize suffering. All procedures for animal care were approved by the Animal Management Committee of Matsumoto Dental University (permit number 292) and performed accordingly.

### *In vitro* osteoclast differentiation assay

Primary murine calvarial cells were isolated from the calvariae of neonates, as previously described [14]. To generate osteoclasts, bone marrow cells were co-cultured for 6‒7 d with calvarial cells in minimal essential media (α-MEM) containing penicillin/streptomycin (100 units and 100 μg/ml, respectively), 2 mM L-glutamine, and 10% fetal bovine serum with or without baricitinib (Chemscene, Monmouth Junction, NJ) in the presence of 10^−8^ M 1,25D_3_ and 10^−6^ M PGE_2_ (both; Wako, Osaka, Japan).

Mice were sacrificed by cervical dislocation, and bone marrow cells were collected by flushing the tibia. Bone marrow macrophages were obtained from cultures treated with M-CSF (50 ng ml^-1^) for 3 d and subsequently cultured with or without baricitinib in the presence of M-CSF and GST-RANKL (200 ng ml^-1^; Oriental Yeast, Tokyo, Japan) for 3‒4 d. These cultures were fixed with 4% paraformaldehyde in PBS and stained for tartrate-resistant acid phosphatase (TRAP) activity, as previously described [14]. TRAP-positive multinuclear cells (TRAP^+^ MNC; more than three nuclei) were counted as osteoclasts.

### Cell viability assay in osteoblasts

Calvaria-derived osteoblasts were enriched in 96-well plates and cultured with indicated doses of baricitinib or vehicle (DMSO). After 24 h of treatment, cell numbers were counted using a cell counting kit-8 (Dojindo, Kumamoto, Japan).

### Quantitative reverse transcription-PCR (qPCR) assay

After reaching confluence, the osteoblasts were cultured for 24 h with 2.5 μM baricitinib or DMSO in the presence or absence of 1,25D_3_ and PGE_2_. The *β-actin*, *RANKL*, *M-CSF*, *Osteoprotegerin* (*OPG*), *Suppressor of cytokine signaling 3* (*Socs3*), *Interleukin-6* (*IL-6*), *IL-11*, and *leukemia inhibitory factor* (*LIF*) transcript abundances were measured by qPCR. Total cellular RNA was extracted from osteoblasts using TRIzol Reagent (Invitrogen, Carlsbad, CA), and 2.5 μg was reverse transcribed from an oligo(dT) primer and a random primer using reverse transcriptase (ReverTra Ace; Toyobo, Osaka, Japan). The PCR (20 μl) contained 5 ng of cDNA, 50 nM of each primer, 10 μl of 2×SYBR Green PCR Master Mix (Applied Biosystems, Foster City, CA), and H_2_O. The amplification was accomplished with a StepOne Plus instrument (Applied Biosystems) programmed for incubations of 20 s at 95°C, followed by 40 cycles of denaturation for 1 s at 95°C and an annealing/extension for 20 s at 60°C. Each primer set was purchased from Takara Bio Inc. (Otsu, Shiga, Japan). Each qPCR included reactions with serially diluted standard cDNA. The transcript copy number in each unknown sample was determined from C_T_ by reference to the appropriate standard curve. Each primer set produced the same single peak melting curve for all of the samples, and a single band with the predicted size was detected by 2% agarose gel electrophoresis. The data were calculated as transcript abundance relative to *β-actin* as the internal standard.

### Immunoblotting assay

Cell lysates preparation and SDS-polyacrylamide gel electrophoresis /immunoblotting analysis were performed according to a standard protocol. Proteins were harvested in cell lysis buffer supplemented with proteinase inhibitor cocktail (Sigma-Aldrich, St. Louis, MO, 1:100) and phosphatase inhibitor cocktail 2 and 3 (Sigma-Aldrich, 1:100). Immunoblotting was performed using the following antibodies; anti-phospho Jak1 (Tyr1022/1023) rabbit IgG (3331; Cell Signaling Technology, Beverly, MA, 1:1000), anti-Jak1 rabbit IgG (3332; Cell Signaling Technology, 1:1000), anti-phospho Jak2 (Tyr1007/1008) rabbit IgG (3776; Cell Signaling Technology, 1:1000), anti-Jak2 rabbit IgG (3230; Cell Signaling Technology, 1:1000), anti-RANKL goat IgG (sc-7628; Santa Cruz Biotechnology, Santa Cruz, CA, 1:1000), anti-phospho Stat3 (Tyr705) rabbit IgG (9145; Cell Signaling Technology, 1:2000), anti-Stat3α rabbit IgG (8768; Cell Signaling Technology, 1:1000), anti-α tubulin mouse IgG (CP06; Calbiochem, San Diego, CA, 1:1000), donkey anti-rabbit IgG-HRP (NA934V; GE Healthcare, Little Chalfont, UK, 1:5000), goat anti-mouse IgG-HRP (170–6516; Bio-Rad Laboratories, Hercules, CA, 1:2000), and donkey anti-goat IgG-HRP (sc-2056; Santa Cruz Biotechnology, 1:5000).

### Mouse cytokine protein array

Osteoblasts and bone marrow cells were enriched in the presence or absence of 1,25D_3_ and PGE_2_ for 3 days, and the supernatants were collected. To determine the presence of various cytokines, we used the proteome profiler mouse XL cytokine array kit (ARY028; R&D Systems, Minneapolis, MN), according to the manufacturer's instructions. The dot blot membranes were analyzed using ImageJ software (National Institutes of Health, Bethesda, MD) and normalized to reference spots. Cytokine spots were averaged, the backgrounds subtracted, and the average values reported for each cytokine.

### Adenovirus-mediated knockdown of Jak1 or Jak2

Short hairpin RNAs (shRNAs) were designed to target mouse Jak1 or Jak2 using an shRNA sequence designing tool published by Takara Bio. The designed sequence was inserted into a pSIREN vector, and then ligated into adenoviral vector pAdenoX-ZsGreen1 (Takara Bio). The linearized vectors were transfected into HEK293T cells to produce an adenovirus, according to the manufacturer’s instructions. Osteoblasts were transduced with virus supernatant for 24 h before treatment with 1,25D_3_ and PGE_2_ for qPCR analysis, or simultaneously for the osteoclast differentiation assay. The target sequences of Jak1 or Jak2 are as follows; sh-Jak1#1; 5’- TCCGAACCGAATCATCACT-3’, sh-Jak1#2; 5’- TGGCGACATTCTCCAAAGA-3’, sh-Jak2#1; 5’-GTGGTATTACGCCTGTGTA-3’, sh-Jak2#2; 5’-CAGGCATGACATACTCTAC-3’.

### Statistical analyses

For cell culture experiments, all experiments were performed in triplicate and similar results were obtained. All values represented the means with the SE. Statistical analysis was performed using Statcel 3 software. Results were analyzed using 1-way ANOVAs. When the 1-way ANOVA showed significance, results were compared using a 2-tailed Student’s *t*-test after Tukey’s correction for multiple comparisons, or using Dunnett’s multiple comparison test. A *P* value of less than 0.05 was considered to be significant.

## Results

**Fig 1:**
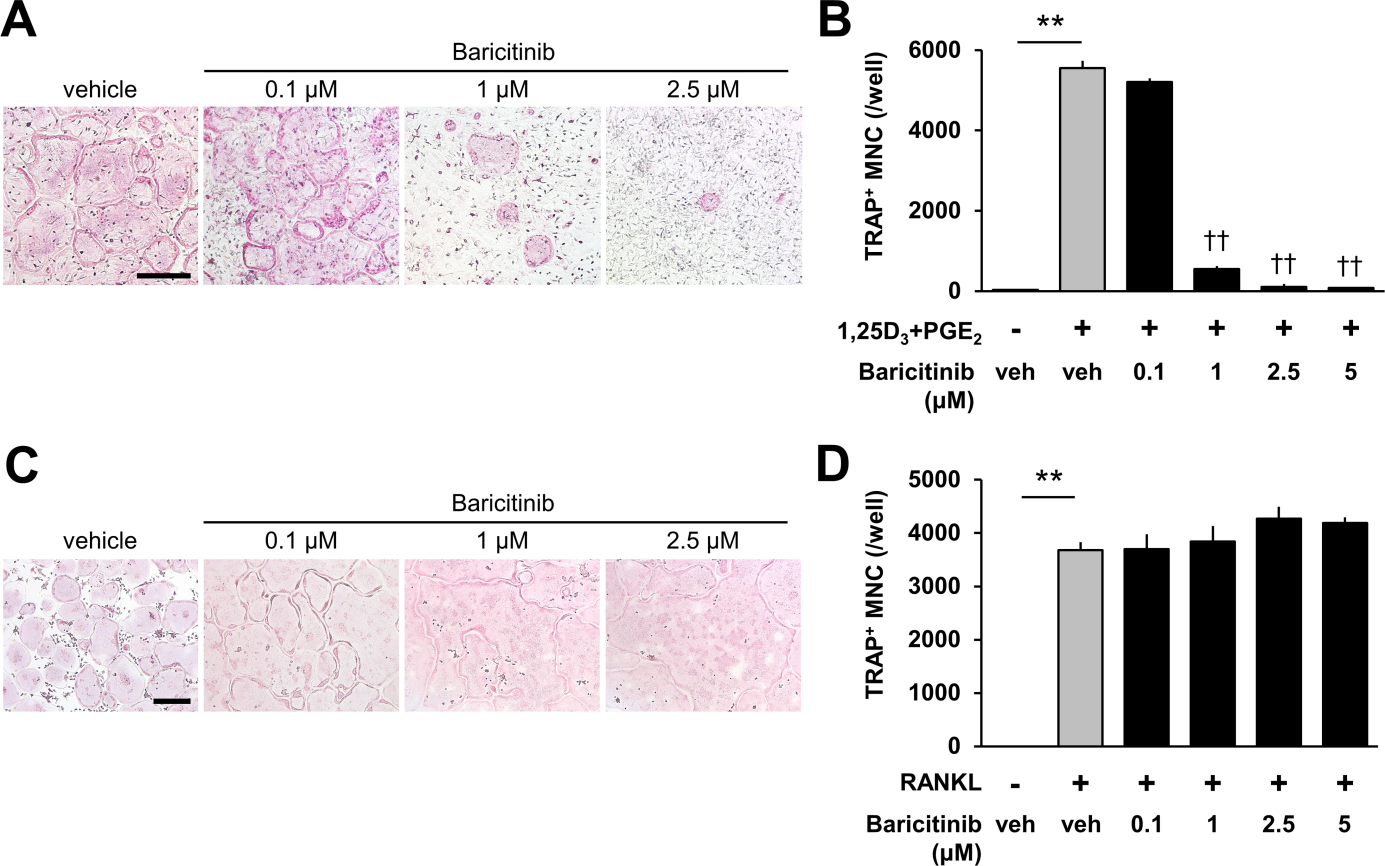
A Jak1/Jak2 inhibitor, baricitinib, suppressed osteoclastogenesis in co-cultures of osteoblasts and bone marrow cells, but not RANKL-induced osteoclastogenesis. **(A, B)** Effects of baricitinib on osteoclast formation in co-cultures of calvaria-derived osteoblasts and bone marrow cells as osteoclast precursors, treated with 10^−8^ M 1,25D_3_ and 10^−6^ M PGE_2_. **(C, D)** Effects of baricitinib on osteoclast formation in bone marrow macrophage cultures, treated with 200 ng ml^−1^ GST-RANKL (+) or untreated (−) in the presence of 50 ng ml^−1^ M-CSF. Micrographs show TRAP staining of osteoclasts. Scale bar, 500 μm. In **B** and **D**: error bars, s.e. (n = 4). **P < 0.01, Student's *t* test. † †*P* < 0.01, Dunnett’s multiple comparisons test (vs vehicle with 1,25D_3_ and PGE_2_).

To examine the effects of baricitinib on osteoclast differentiation *in vitro*, mouse bone marrow cells were co-cultured with calvaria-derived osteoblasts in the presence of 10^−8^ M 1,25D_3_ and 10^−6^ M PGE_2_ (Fig 1A and 1B). Mouse bone marrow cells efficiently differentiated into TRAP^+^-multinuclear osteoclasts in the presence of 1,25D_3_ and PGE_2_ within 7 d, while the treatment with baricitinib suppressed TRAP^+^-osteoclast formation in a dose-dependent manner. Next, we examined effects of baricitinib on RANKL-induced osteoclast formation (Fig 1C and 1D). Bone marrow macrophages as osteoclast precursors were cultured in the presence of M-CSF and RANKL with/without baricitinib. Unlike co-cultures, baricitinib did not inhibit RANKL-induced osteoclast differentiation (Fig 1D), and giant osteoclasts were frequently observed in the cultures with baricitinib (Fig 1C). These results indicated that baricitinib acted on osteoblasts and then inhibited osteoclastogenesis.

**Fig 2:**
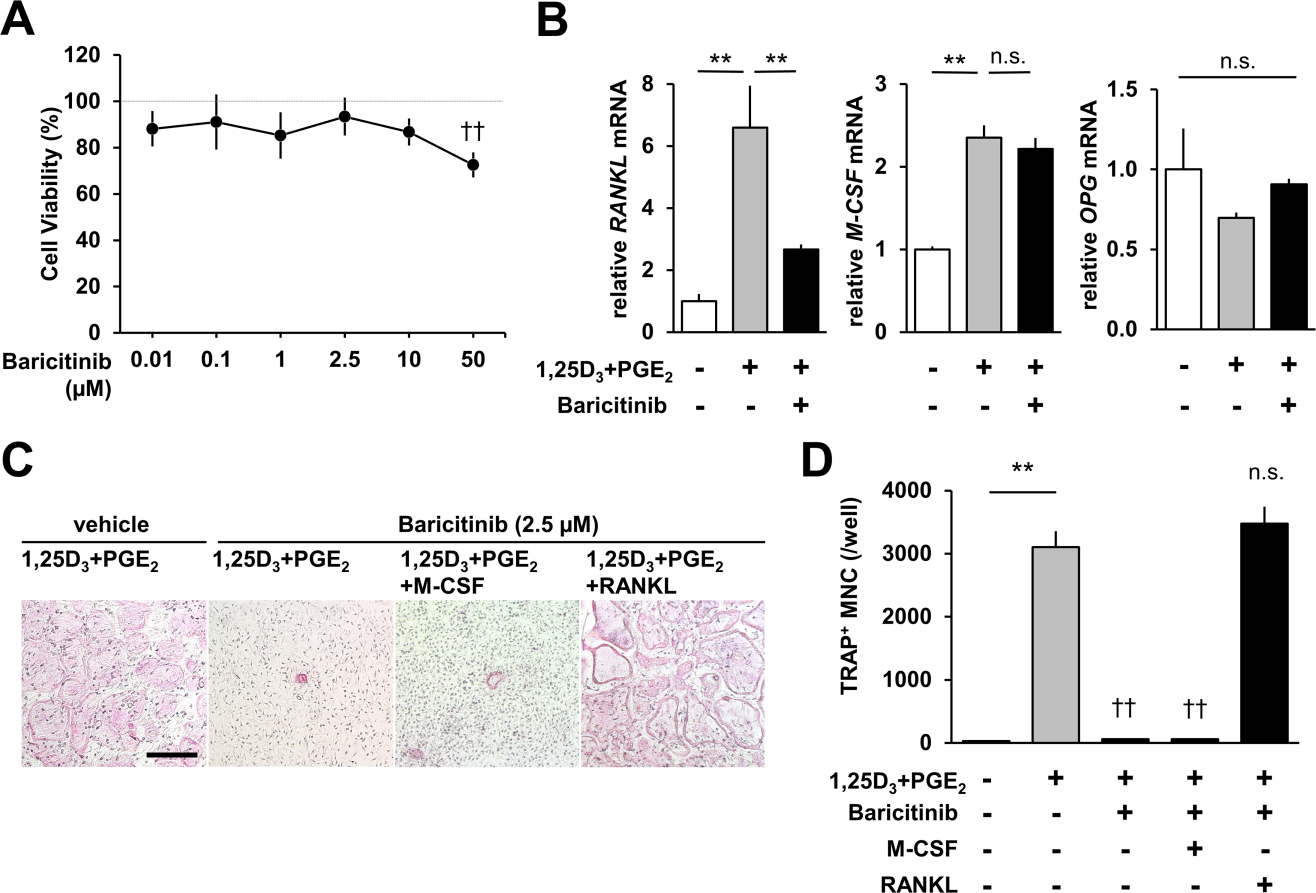
Baricitinib inhibits osteoclastogenesis by suppressing RANKL expression in osteoblasts. **(A)** The dose-response curve of viability of osteoblasts treated with baricitinib for 24 h. 100% was determined as the cell numbers when treated with vehicle (DMSO). **(B)** Effects of 2.5 μM baricitinib on expression of *RANKL*, *M-CSF*, and *OPG* mRNAs in osteoblasts in the presence of 1,25D_3_ and PGE_2_. **(C, D)** 200 ng ml^-1^ GST-RANKL, but not 50 ng ml^-1^ M-CSF, completely cancelled effects of 2.5 μM baricitinib on osteoclast formation in co-cultures. Micrographs show TRAP staining of osteoclasts. Scale bar, 500 μm. In **A, B,** and **D**: error bars, s.e. (n = 6 (A), n = 3–4 (B, D)). **P < 0.01, Student’s *t* test. † †*P* < 0.01, n.s. not significant, Dunnett’s multiple comparisons test (vs vehicle **(A)** or vehicle with 1,25D_3_ and PGE_2_ **(D)**).

We examined the effects of baricitinib on the cell viability of osteoblasts (Fig 2A). Doses of 0.01‒10 μM baricitinib did not affect the viability of osteoblasts, and a dose of 2.5 μM was used in subsequent experiments. We next assessed the effects of baricitinib on the ability of osteoblasts to induce osteoclast differentiation. 1,25D_3_ and PGE_2_ up-regulated the expression of *RANKL* and *M-CSF* mRNA in primary osteoblasts and slightly down-regulated the expression of *Osteoprotegerin* (*OPG*) mRNA (Fig 2B). Then, baricitinib treatment significantly inhibited 1,25D_3_ and PGE_2_-induced up-regulation of *RANKL* mRNA, but did not alter the expression of *M-CSF* and *OPG*. Given our findings that baricitinib treatment led to a decrease of *RANKL* up-regulation, we investigated the effects of RANKL on osteoclastogenesis under baricitinib treatment. Suppression of osteoclastogenesis by baricitinib treatment was rescued when recombinant RANKL, but not M-CSF, was added to the co-cultures (Fig 2C and 2D). Thus, baricitinib inhibited osteoclastogenesis by suppressing RANKL expression in osteoblasts.

**Fig 3:**
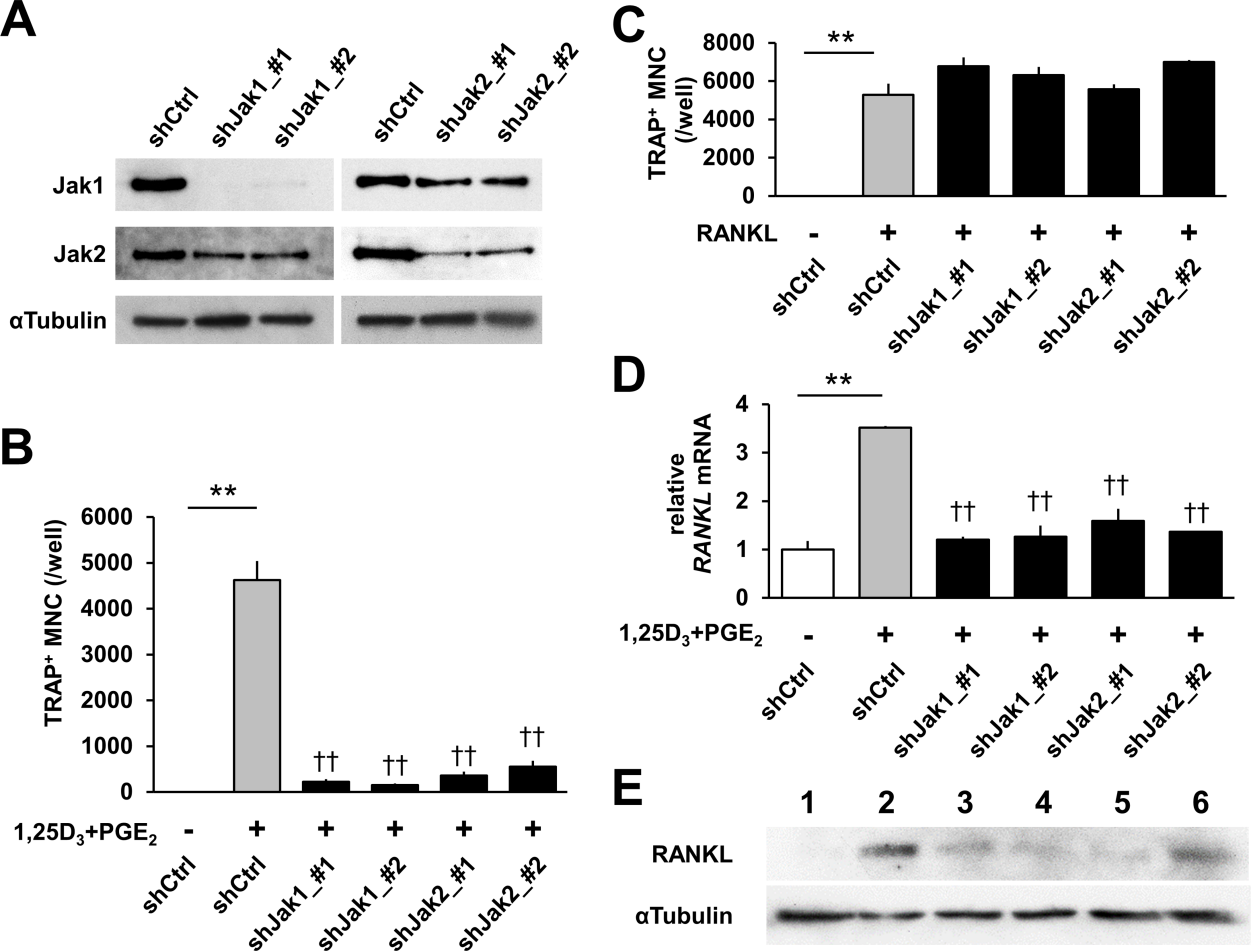
shRNA-mediated knockdown of Jak1 or Jak2 also suppressed expression of RANKL in osteoblasts. **(A)** Immunoblot images of adenovirus-mediated knockdown of murine Jak1 or Jak2. **(B, C)** Effects of shJak1_#1, #2, shJak2_#1 or #2 on osteoclast formation in co-cultures of osteoblasts and bone marrow cells treated with 1,25D_3_ and PGE_2_ **(B)** or in bone marrow macrophage cultures treated with RANKL and M-CSF **(C)**. **(D, E)** Effects of shJak1_#1, #2, shJak2_#1 or #2 on expression of RANKL mRNAs **(D)** and proteins **(E)** in osteoblasts. Osteoblasts were transfected with shCtrl (lane 1, lane 2), shJak1_#1 (lane 3), shJak1_#2 (lane 4), shJak2_#1 (lane 5) or shJak2_#2 (lane 6) and treated with (lane 2–6) or without (lane 1) 1,25D_3_ and PGE_2_ for 24 h. In **B–D**: error bars, s.e. (n = 3–4). **P < 0.01, Student's *t* test. † †*P* < 0.01, Dunnett’s multiple comparisons test (vs shCtrl with 1,25D_3_ and PGE_2_). Original immunoblot images are shown in S1 Fig.

To assess whether Jak1 or Jak2 induces RANKL expression in osteoblasts, we silenced Jak1 or Jak2 expression by adenovirus-mediated expression of shRNA against Jak1 or Jak2. The knockdown efficiency and specificity were verified by immunoblotting (Fig 3A). Although bone marrow cells differentiated into TRAP^+^-multinuclear osteoclasts in control shRNA, either Jak1 or Jak2 silencing strongly decreased osteoclast formation in co-cultures (Fig 3B). In contrast, shRNA-mediated knockdown of Jak1 or Jak2 in bone marrow macrophages failed to reduce RANKL-induced osteoclastogenesis (Fig 3C). In addition, up-regulated expression of RANKL elicited by 1,25D_3_ and PGE_2_ was cancelled by shJak1 or shJak2 (Fig 3D and 3E). These data suggested that osteoblasts require Jak1 as well as Jak2 in 1,25D_3_ and PGE_2_-induced RANKL expression.

**Fig 4:**
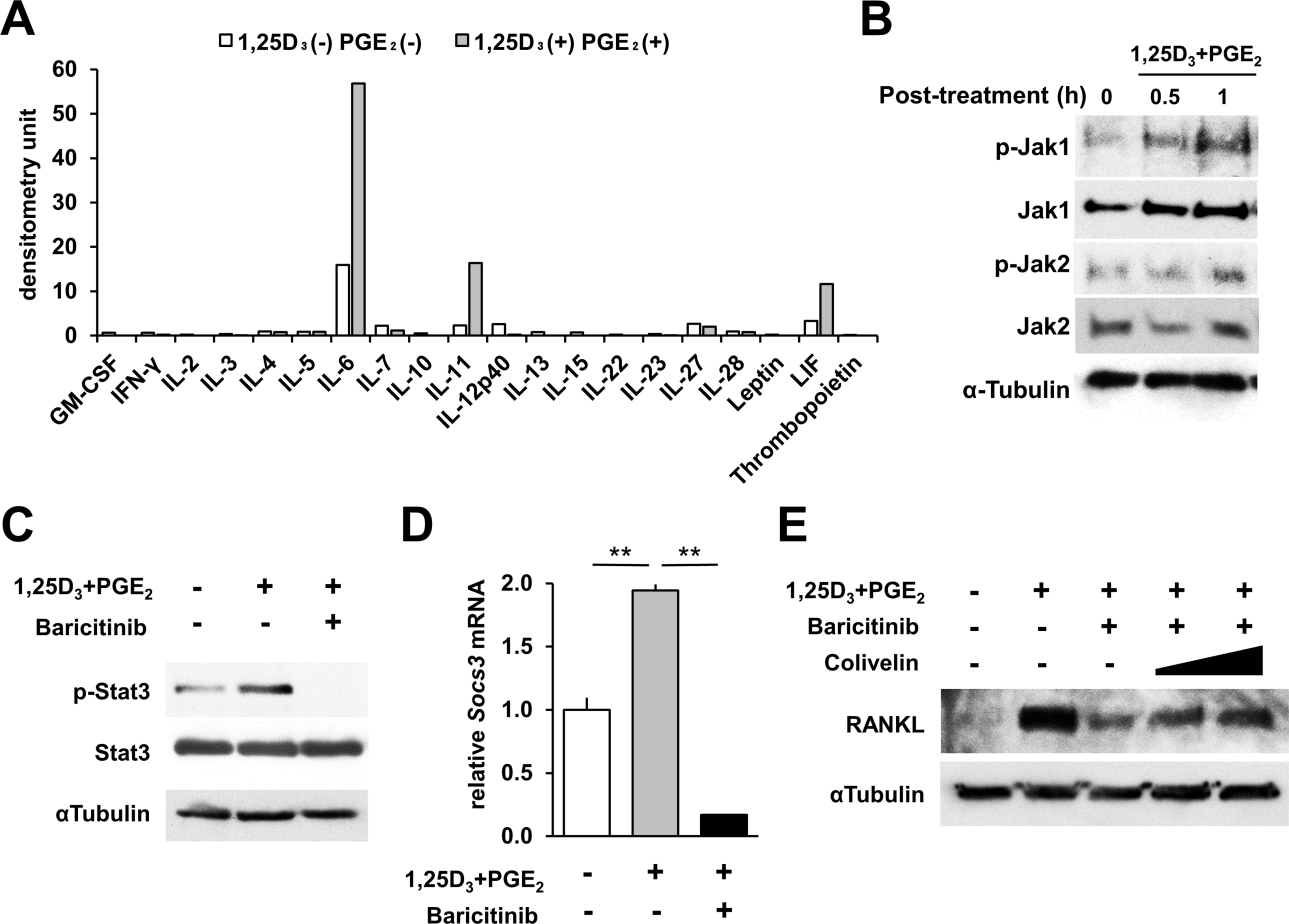
1,25D_3_ and PGE_2_ stimulate secretion of IL-6 family cytokines in co-cultures and activate Jak1-Jak2/Stat3 signaling in osteoblasts. **(A)** Densitometric data of cytokine protein array in co-cultured medium of osteoblasts and bone marrow cells in the presence or absence of 1,25D_3_ and PGE_2_. Two independent experiments were performed, and a representative result is shown. **(B, C)** Osteoblasts were stimulated by 1,25D_3_ and PGE_2_ for 0.5 **(B)** or 1 h **(B, C)**, and phosphorylation of Jak1, Jak2, and Stat3 were determined by immunoblotting. **(D)** Effects of 2.5 μM baricitinib on expression of *Socs3* mRNA in osteoblasts in the presence of 1,25D_3_ and PGE_2_. **(E)** An activator of Stat3, colivelin (Santa Cruz Biotechnology; 0.1 or 1 µM), rescued baricitinib-induced RANKL down-regulation in osteoblast cultures. error bars, s.e. (n = 3). **P < 0.01, Student’s *t* test. Original immunoblot images are shown in S1 Fig.

Further, we investigated whether 1,25D_3_ and PGE_2_ alter the expression of type I/II cytokines with a cytokine array. We enriched osteoblasts and bone marrow derived cells for 3 days, and then examined the supernatant. The results revealed that secretion of three interleukin-6 (IL-6) family cytokines, IL-6, IL-11, and leukemia inhibitory factor (LIF), were stimulated by 1,25D_3_ and PGE_2_ (Fig 4A). These cytokines bind to their receptor complexes that share the signal transducer glycoprotein 130 (gp130) and subsequently activate Jaks. This activation enables Jaks to recruit and phosphorylate the signal transducers and activators of transcription 3 (Stat3) [15]. Indeed, Jak1 and Jak2 in osteoblasts were phosphorylated by treatment with 1,25D_3_ and PGE_2_ for 1 h (Fig 4B). In addition, 1,25D_3_ and PGE_2_ phosphorylated Stat3 in osteoblasts (Fig 4C) and up-regulated *suppressor of cytokine signaling 3* (*Socs3*) mRNA, which is a target gene of Stat3 (Fig 4D). Jak1/2 inhibitor baricitinib inhibited the activations. Immunoblot analysis further revealed that an activator of Stat3, colivelin, rescued baricitinib-induced RANKL suppression in osteoblasts (Fig 4E). Finally, we confirmed that IL-6 family cytokine LIF induced osteoclast formation in co-cultures and treatment with baricitinib significantly inhibited the osteoclast formation (S2 Fig). These results suggest that baricitinib inhibits gp130/Jak1-2/Stat3 signal transduction, which in turn suppresses RANKL expression in co-cultures treated with 1,25D_3_ and PGE_2_.

## Discussion

Baricitinib (formerly INCB-28050), which was co-developed by Incyte and Lilly, has been shown to be a potent and selective inhibitor of Jak1 and Jak2. Baricitinib has shown efficacy in rats with collagen-induced arthritis or anti-collagen antibody-induced arthritis [16]. In human RA, baricitinib halts radiographic disease progression, with significant differences in erosions and joint-space narrowing [17]. The mechanism by which baricitinib prevents bone destruction in arthritis has not yet been fully investigated. The present study demonstrates a possible pathway by which baricitinib inhibits RANKL expression in osteoblasts, thereby suppressing osteoclastogenesis. Thus, Jak1 and Jak2 represent novel therapeutic targets for bone metabolic diseases.

1,25D_3_ and PGE_2_ induce RANKL expression in osteoblasts *via* vitamin D receptors and PGE_2_ receptors 2 and 4, respectively [18, 19]. However, osteoclastogenesis induced by 1,25D_3_ and PGE_2_ is suppressed by anti-gp130 neutralization antibody [20]; therefore, the function of 1,25D_3_ and PGE_2_ as an inducer of osteoclastogenesis is considered to, at least partly, depend on the gp130 signaling pathway. This study found that 1,25D_3_ and PGE_2_ stimulated IL-6, IL-11, and LIF secretion in co-cultures. 1,25D_3_ and PGE_2_ up-regulated *IL-6*, *IL-11*, and *LIF* mRNA expressions in osteoblasts (S2 Fig) and led to activation of both Jak1 and Jak2 and subsequent phosphorylation of Stat3 in osteoblast cultures. These findings indicate that secreted IL-6, IL-11, and LIF in co-cultures are, at least partially, derived from osteoblasts and have effects on RANKL expression in an autocrine manner. Thus, IL-6, IL-11, and/or LIF play a key role in osteoclast formation induced by 1,25D_3_ and PGE_2_ in a co-culture of osteoblasts and bone marrow cells.

Jaks are involved in various cellular events, and which Jak is involved in these events depends on the cell [15]. In fibrosarcoma-derived cells, IL-6 signaling depends on the presence of Jak1 [21]. In osteoblasts, however, it has not been determined whether Jaks are interchangeable, or whether there is a hierarchy in IL-6 family cytokine signaling. In this study, adenovirus-mediated knockdown of Jak1 or Jak2 strongly reduced 1,25D_3_ and PGE_2_-induced *RANKL* expression in osteoblasts and suppressed osteoclastogenesis in co-cultures. These results indicate that osteoblasts require both Jak1 and Jak2 in the IL-6 family signaling pathway in a non-redundant manner, and that Jak1 and Jak2 form a hetero-dimer or that there is a hierarchy in the signaling pathway in osteoblasts.

Previous studies described that tofacitinib (a pan-Jak inhibitor) did not inhibit RANKL-induced osteoclastogenesis in bone marrow macrophage cultures in the absence of osteoblastic cells [10, 11]. Our data using baricitinib were compatible with these previous results. We also found large osteoclasts in bone marrow macrophage cultures in the presence of RANKL and M-CSF with baricitinib. Impaired LIF/LIF receptor signaling led to increased survival and fusion of osteoclast precursors *via* the enhancement of Bcl-2 expression [22, 23]. The number and size of osteoclasts were increased in *LIF*-mutant newborn mice [24]. Furthermore, osteoclasts were larger in bone marrow macrophage cultures prepared from cathepsin-K-driven gp130-deficient mice [25]. Based on these findings, we speculate that baricitinib would inhibit LIF/gp130 signaling; therefore, big osteoclasts would be formed.

*In vivo* roles of the gp130/JAK pathway in osteoclastogenesis remain controversial [26]. When gp130 was deleted in the entire osteoblast lineage, the osteoclast number did not significantly differ between knockout mice and controls [27]. This finding suggests that the stimulatory actions of IL-6 family cytokines on RANKL expression and osteoclast generation by the osteoblast lineage may not be largely involved in physiological bone remodeling. On the other hand, several studies have mentioned the contribution of the gp130/JAK pathway to pathological bone resorption. Global IL-6-null mice failed to exhibit increased osteoclastogenesis in response to ovariectomy [28] or inflammatory arthritis [29]. Since IL-6 family cytokines can also stimulate bone formation [30–36], inhibition of the IL-6 family/gp130/Jak pathway would be effective for excessive bone resorption, but not for the physiological state.

Taken together, the present study revealed the role of Jak1 and Jak2 in osteoclastogenesis *in vitro*. In a co-culture of osteoblasts and bone marrow cells, 1,25D_3_ and PGE_2_ simulate secretion of IL-6, IL-11, and LIF and induce expression of RANKL in osteoblasts *via* the gp130/Jak signaling pathway. This pathway depends on the presence of both Jak1 and Jak2; therefore, a selective Jak1/2 inhibitor, baricitinib, inhibits osteoclast formation. Thus, baricitinib is a potential therapeutic agent to prevent bone resorption.

## Supporting information

S1 Fig Original images of immunoblot analyses presented in Figs 3A, 3E, 4B, 4C and 4E. (PDF)

S2 Fig Baricitinib inhibits LIF-induced osteoclastogenesis in the co-culture. Effects of 2.5 μM baricitinib on osteoclast formation in co-cultures of calvaria-derived osteoblasts and bone marrow cells as osteoclast precursors, treated with 10^2^ units ml^-1^ LIF (ESGRO^®^, Merck Millipore). error bars, s.e. (n = 4). **P < 0.01, Student's *t* test.
(PDF)

S3 Fig 1,25D_3_ and PGE_2_ up-regulated the expression of *IL-6*, *IL-11*, and *LIF* mRNA in osteoblasts. Primary osteoblasts were cultured for 24 h in the presence or absence of 10^−8^ M 1,25D_3_ and 10^−6^ M PGE_2_. Total cellular RNA was extracted from osteoblasts, and 2.5 μg was reverse transcribed. Then, qPCR analysis was performed. error bars, s.e. (n = 3). *P < 0.05, **P < 0.01, Student's *t* test.
(PDF)
