# Supplementary material for: A Jak1/2 inhibitor, baricitinib, inhibits osteoclastogenesis by suppressing RANKL expression in osteoblasts in vitro
Source: PLoS One. 2017 Jul 14;12(7):e0181126. doi: 10.1371/journal.pone.0181126 (PMC5510865; doi:10.1371/journal.pone.0181126)
Supplement: S1 Fig — (PDF) [file pone.0181126.s001.pdf]

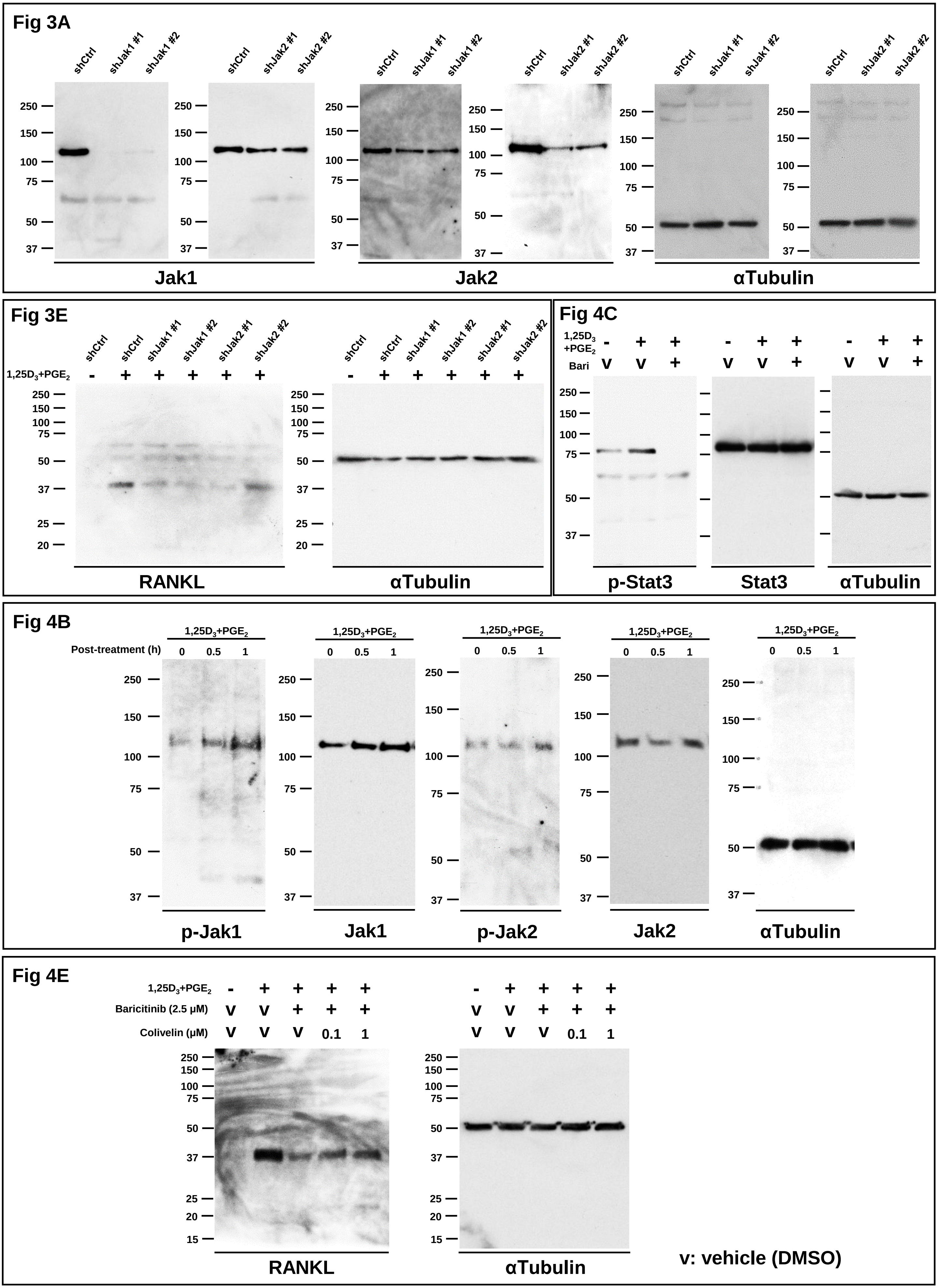

S1 Fig. Original images of immunoblot analyses presented in Fig 3A, 3E, 4B, 4C, and 4E.
