# Supplementary material for: A Jak1/2 inhibitor, baricitinib, inhibits osteoclastogenesis by suppressing RANKL expression in osteoblasts in vitro
Source: PLoS One. 2017 Jul 14;12(7):e0181126. doi: 10.1371/journal.pone.0181126 (PMC5510865; doi:10.1371/journal.pone.0181126)
Supplement: S2 Fig — Effects of 2.5 μM baricitinib on osteoclast formation in co-cultures of calvaria-derived osteoblasts and bone marrow cells as osteoclast precursors, treated with 102 units ml-1 LIF (ESGRO®, Merck Millipore). error bars, s.e. (n = 4). **P < 0.01, Student's t test. (PDF) [file pone.0181126.s002.pdf]

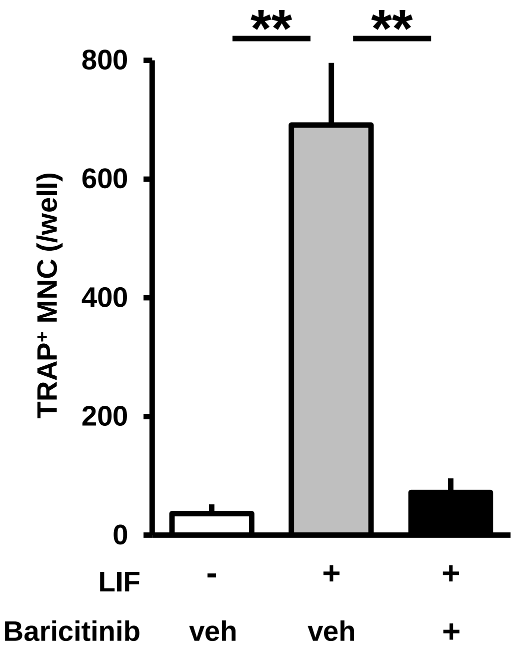

S2 Fig.
Baricitinib inhibits LIF-induced osteoclastogenesis in the co-culture.
Effects of 2.5 μM baricitinib on osteoclast formation in co-cultures of calvaria-derived osteoblasts and bone marrow cells as osteoclast precursors, treated with 10^2 units ml^{-1} LIF (ESGRO®, Merck Millipore). error bars, s.e. (n = 4). **P < 0.01, Student's t test.
