# Supplementary material for: A Jak1/2 inhibitor, baricitinib, inhibits osteoclastogenesis by suppressing RANKL expression in osteoblasts in vitro
Source: PLoS One. 2017 Jul 14;12(7):e0181126. doi: 10.1371/journal.pone.0181126 (PMC5510865; doi:10.1371/journal.pone.0181126)
Supplement: S3 Fig — Primary osteoblasts were cultured for 24 h in the presence or absence of 10−8 M 1,25D3 and 10−6 M PGE2. Total cellular RNA was extracted from osteoblasts, and 2.5 μg was reverse transcribed. Then, qPCR analysis was performed. error bars, s.e. (n = 3). *P < 0.05, **P < 0.01, Student's t test. (PDF) [file pone.0181126.s003.pdf]

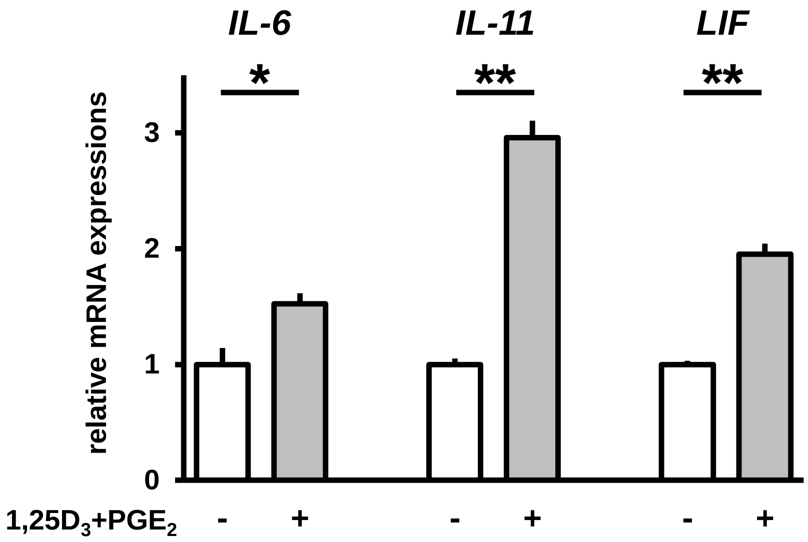

S3 Fig.
$1,25D_3$ and PGE_2 up-regulated the expression of *IL-6*, *IL-11,* and *LIF* mRNA in osteoblasts.
Primary osteoblasts were cultured for 24 h in the presence or absence of 10^{-8} M $1,25D_3$ and 10^{-6} M PGE_2.
Total cellular RNA was extracted from osteoblasts, and 2.5 μg was reverse transcribed.
Then, qPCR analysis was performed. error bars, s.e. (n = 3). *$P < 0.05$, **$P < 0.01$, Student's *t* test.
